# Explainable machine learning in outcome prediction of high-grade aneurysmal subarachnoid hemorrhage

**DOI:** 10.18632/aging.205621

**Published:** 2024-03-01

**Authors:** Lei Shu, Hua Yan, Yanze Wu, Tengfeng Yan, Li Yang, Si Zhang, Zhihao Chen, Qiuye Liao, Lu Yang, Bing Xiao, Minhua Ye, Shigang Lv, Miaojing Wu, Xingen Zhu, Ping Hu

**Affiliations:** 1 Department of Neurosurgery, The Second Affiliated Hospital, Jiangxi Medical College, Nanchang University, Nanchang 330006, Jiangxi, China; 2 Jiangxi Key Laboratory of Neurological Tumors and Cerebrovascular Diseases, Nanchang 330006, Jiangxi, China; 3 Jiangxi Health Commission Key Laboratory of Neurological Medicine, Nanchang 330006, Jiangxi, China; 4 Institute of Neuroscience, Nanchang University, Nanchang 330006, Jiangxi, China; 5 Department of Emergency, Affiliated Hospital of Panzhihua University, Panzhihua 617000, Sichuan, China

**Keywords:** aneurysmal subarachnoid hemorrhage, high-grade, explainable machine learning, SHapley additive exPlanations, prognosis prediction

## Abstract

Objective: Accurate prognostic prediction in patients with high-grade aneruysmal subarachnoid hemorrhage (aSAH) is essential for personalized treatment. In this study, we developed an interpretable prognostic machine learning model for high-grade aSAH patients using SHapley Additive exPlanations (SHAP).

Methods: A prospective registry cohort of high-grade aSAH patients was collected in one single-center hospital. The endpoint in our study is a 12-month follow-up outcome. The dataset was divided into training and validation sets in a 7:3 ratio. Machine learning algorithms, including Logistic regression model (LR), support vector machine (SVM), random forest (RF), and extreme gradient boosting (XGBoost), were employed to develop a prognostic prediction model for high-grade aSAH. The optimal model was selected for SHAP analysis.

Results: Among the 421 patients, 204 (48.5%) exhibited poor prognosis. The RF model demonstrated superior performance compared to LR (AUC = 0.850, 95% CI: 0.783-0.918), SVM (AUC = 0.862, 95% CI: 0.799-0.926), and XGBoost (AUC = 0.850, 95% CI: 0.783-0.917) with an AUC of 0.867 (95% CI: 0.806-0 .929). Primary prognostic features identified through SHAP analysis included higher World Federation of Neurosurgical Societies (WFNS) grade, higher modified Fisher score (mFS) and advanced age, were found to be associated with 12-month unfavorable outcome, while the treatment of coiling embolization for aSAH drove the prediction towards favorable prognosis. Additionally, the SHAP force plot visualized individual prognosis predictions.

Conclusions: This study demonstrated the potential of machine learning techniques in prognostic prediction for high-grade aSAH patients. The features identified through SHAP analysis enhance model interpretability and provide guidance for clinical decision-making.

## INTRODUCTION

Subarachnoid hemorrhage caused by ruptured intracranial aneurysms (aSAH) is a global health concern due to its significant impact on mortality and long-term disability rates [1]. Approximately 35% of patients succumb to severe cerebrovascular injury in the initial weeks [2]. Among the survivors, a considerable number experience disability. High-grade aSAH is associated with profound neurological consequences resulting from a combination of direct blood-induced damage, secondary vasospasm, and delayed cerebral ischemia [3]. Currently, the assessment of aSAH severity and prediction of clinical outcomes rely on neurological examinations and neuroimaging studies; however, estimating high-grade SAH patients can be challenging due to the frequent administration of sedatives and analgesics during their management [3]. A timely and accurate diagnosis of high-grade SAH is pivotal for instituting appropriate therapeutic interventions. Hence, there is an urgent demand for a functional prediction model to aid in the treatment and evaluation of patients with high-grade SAH.

In recent years, numerous prediction models have been extensively developed to forecast the clinical outcomes in high-grade aSAH patients [4]. However, the majority of these models rely on conventional algorithms with limited clinical features. Recent advancements in artificial intelligence have led to significant breakthroughs in medical machine learning (ML) [5–7], and these models have demonstrated promising discrimination capabilities. In a recent study, a support vector machine (SVM) model was constructed utilizing high-throughput metabolomics data to identify potential biomarkers and targets for the diagnosis and treatment of colorectal cancer, and the model achieved an AUC of 0.985 [8]. Nevertheless, due to the inherent “black box” nature of ML algorithms that lack transparency and explanatory research, elucidating the prediction process within the model becomes a challenge [9]. Shapley Additive Explanations (SHAP) is a novel game theory-based approach in explainable ML introduced by Lundberg and Lee [10], it can well solve the issue of inexplicability by providing a solution for better understanding and interpreting complex models, and this method allows for representing the contribution of each feature to the outcome. Yagin et al. proposed an explainable artificial intelligence model to predict COVID-19 using metagenomic next-generation sequencing (mNGS) data, and the model allowed physicians to enhance their comprehension of the decision-making process in COVID-19 genomic prediction [11]. Another study developed a XGBoost model combined with SHAP to effectively predict the 3-year all-cause mortality in coronary heart disease and heart failure patients. The model offers clear explanations for individualized risk predictions, aiding doctors in understanding the impact of key features [12]. These researches showed explainable machine learning holds great promise in assisting physicians in intuitively grasping the influence of key features in models. This aids clinicians in gaining a deeper understanding of decisions made for disease severity assessment.

Therefore, this study aims to develop and validate an explainable ML model to predict 12-month outcomes in patients with high-grade aSAH. Besides, the SHAP values of each feature were analyzed to elucidate the overall prediction process. This effort will contribute to the development of explainable and personalized predictive models for prognosis in high-grade aSAH, marking a substantial advancement for the application of machine learning in the field of medicine.

## RESULTS

### Baseline characteristics

**Figure 1:**
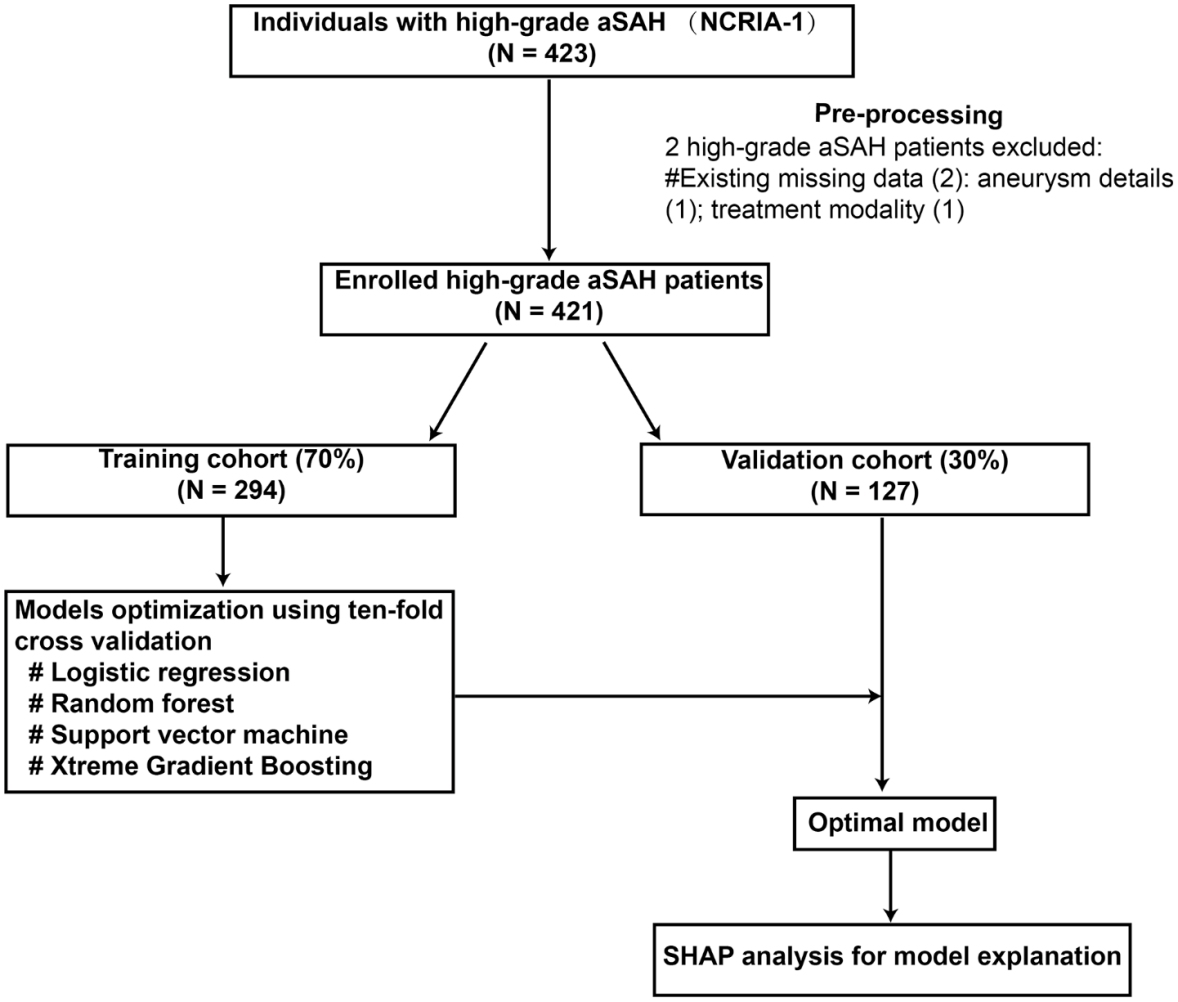
The flowchart of this study.

Among a total of 421 patients with high-grade aSAH, 204 patients suffered poor outcomes in our final cohort. The detailed baseline characteristic of the patients was represented in Table 1. The mean age was 62 (range: 54, 69), and there were 259 (61.5%) female patients in the cohort. The poor outcome group had an older age (P < 0.001), higher rate of coil treatment (P < 0.001), higher rates of hypertension (P < 0.05), higher World Federation of Neurosurgical Societies (WFNS) grade (P < 0.001), and higher modified Fisher score (mFS) (P < 0.001), as well as larger aneurysm length size (P < 0.05) than favorable outcome. Table 2 presents the baseline characteristics of the training set and validation set. And The flowchart of our study is shown in Figure 1.

**Table 1:** Baseline characteristics in patients with high-grade aSAH.

	**Overall**	**Favorable outcome**	**Poor outcome**	**P**
n	421	217	204	
Age (median [IQR])	62.00 [54.00, 69.00]	59.00 [51.00, 65.00]	65.00 [56.00, 71.00]	**<0.001**
Female (%)	259 (61.5)	140 (64.5)	119 (58.3)	0.229
Treatment coiling (%)	283 (67.2)	168 (77.4)	115 (56.4)	**<0.001**
Hypertension (%)	231 (54.9)	108 (49.8)	123 (60.3)	**0.038**
Diabetes (%)	24 (5.7)	8 (3.7)	16 (7.8)	0.104
CHD (%)	9 (2.1)	6 (2.8)	3 (1.5)	0.562
Smoking (%)	39 (9.3)	25 (11.5)	14 (6.9)	0.139
Drinking (%)	20 (4.8)	11 (5.1)	9 (4.4)	0.930
Anticoagulant (%)	15 (3.6)	9 (4.1)	6 (2.9)	0.686
location (%)				0.050
ACA	18 (4.3)	11 (5.1)	7 (3.4)	
MCA	108 (25.7)	48 (22.1)	60 (29.4)	
ICA	16 (3.8)	6 (2.8)	10 (4.9)	
PCA	10 (2.4)	5 (2.3)	5 (2.5)	
ACoA	120 (28.5)	57 (26.3)	63 (30.9)	
PCoA	115 (27.3)	65 (30.0)	50 (24.5)	
Other	34 (8.1)	25 (11.5)	9 (4.4)	
Aneurysm multiple (%)	71 (16.9)	29 (13.4)	42 (20.6)	0.065
Mean aneurysm size				
Length (median [IQR])	5.00 [3.50, 6.60]	4.80 [3.30, 6.20]	5.25 [3.70, 7.00]	**0.027**
Width (median [IQR])	4.00 [3.00, 5.40]	4.00 [3.10, 5.20]	4.00 [3.00, 5.60]	0.982
Neck (median [IQR])	3.50 [2.70, 4.40]	3.50 [2.60, 4.50]	3.50 [2.70, 4.40]	0.850
WFNS (%)				**<0.001**
1	1 (0.2)	1 (0.5)	0 (0.0)	
2	95 (22.6)	77 (35.5)	18 (8.8)	
3	16 (3.8)	10 (4.6)	6 (2.9)	
4	171 (40.6)	104 (47.9)	67 (32.8)	
5	138 (32.8)	25 (11.5)	113 (55.4)	
mFS (%)				**<0.001**
1	10 (2.4)	7 (3.2)	3 (1.5)	
2	66 (15.7)	54 (24.9)	12 (5.9)	
3	147 (34.9)	87 (40.1)	60 (29.4)	
4	198 (47.0)	69 (31.8)	129 (63.2)	

Abbreviations: CHD, coronary heart disease; WFNS, World Federation of Neurological Societies; mFS, modified Fisher Scale; ACA, anterior cerebral artery; MCA, middle cerebral artery; ICA, internal cerebral artery; PCA, posterior cerebral artery; ACoA, anterior communicating artery; PCoA, posterior communicating artery.

**Table 2:** Baseline characteristics of the training set and validation set.

	**Overall**	**Training**	**Validation**	**P**
n	421	294	127	
Age (median [IQR])	62.00 [54.00, 69.00]	63.00 [55.00, 69.00]	59.00 [51.00, 67.00]	0.014
Female (%)	259 (61.5)	175 (59.5)	84 (66.1)	0.241
Treatment coiling (%)	283 (67.2)	206 (70.1)	77 (60.6)	0.075
Hypertension (%)	231 (54.9)	167 (56.8)	64 (50.4)	0.038
Diabetes (%)	24 (5.7)	17 (5.8)	7 (5.5)	1.000
CHD (%)	9 (2.1)	7 (2.4)	2 (1.6)	0.875
Smoking (%)	39 (9.3)	34 (11.6)	5 (3.9)	0.022
Drinking (%)	20 (4.8)	17 (5.8)	3 (2.4)	0.206
Anticoagulant (%)	15 (3.6)	9 (3.1)	6 (4.7)	0.576
location (%)				0.104
ACA	18 (4.3)	14 (4.8)	4 (3.1)	
MCA	108 (25.7)	68 (23.1)	40 (31.5)	
ICA	16 (3.8)	10 (3.4)	6 (4.7)	
PCA	10 (2.4)	9 (3.1)	1 (0.8)	
ACoA	120 (28.5)	90 (30.6)	30 (23.6)	
PCoA	115 (27.3)	84 (28.6)	31 (24.4)	
Other	34 (8.1)	19 (6.5)	15 (11.8)	
Aneurysm multiple (%)	71 (16.9)	47 (16.0)	24 (18.9)	0.555
Mean aneurysm size				
Length (median [IQR])	5.00 [3.50, 6.60]	5.00 [3.50, 6.60]	5.10 [3.50, 6.70]	0.924
Width (median [IQR])	4.00 [3.00, 5.40]	3.90 [3.00, 5.30]	4.10 [3.10, 5.55]	0.397
Neck (median [IQR])	3.50 [2.70, 4.40]	3.30 [2.50, 4.40]	3.70 [3.00, 4.65]	0.039
WFNS (%)				0.676
1	1 (0.2)	1 (0.3)	0 (0.0)	
2	95 (22.6)	63 (21.4)	32 (25.2)	
3	16 (3.8)	10 (3.4)	6 (4.7)	
4	171 (40.6)	125 (42.5)	46 (36.2)	
5	138 (32.8)	95 (32.3)	43 (33.9)	
mFS (%)				0.864
1	10 (2.4)	6 (2.0)	4 (3.1)	
2	66 (15.7)	45 (15.3)	21 (16.5)	
3	147 (34.9)	102 (34.7)	45 (35.4)	
4	198 (47.0)	141 (48.0)	57 (44.9)	
Poor outcome (%)	204 (48.5)	141 (48.0)	63 (49.6)	0.838

Abbreviations: CHD, coronary heart disease; WFNS, World Federation of Neurological Societies; mFS, modified Fisher Scale; ACA, anterior cerebral artery; MCA, middle cerebral artery; ICA, internal cerebral artery; PCA, posterior cerebral artery; ACoA, anterior communicating artery; PCoA, posterior communicating artery.

### Model development and validation

**Figure 2:**
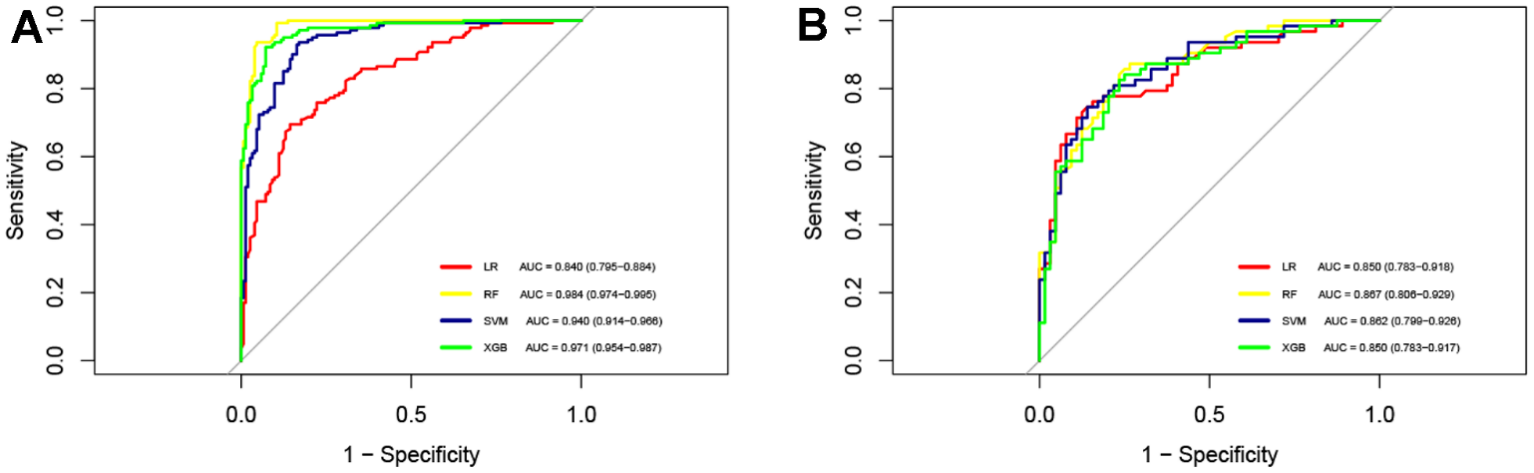
**ROC curves for four machine learning models.** (**A**) AUCs of four machine learning models in the training cohort; (**B**) AUCs of four machine learning models in the test cohort. ROC, receiver operating characteristic curve; AUC, area under the curve; LR, logistic regression; RF, random forest; SVM, support vector machine; XGB, extreme gradient boosting.

**Figure 3:**
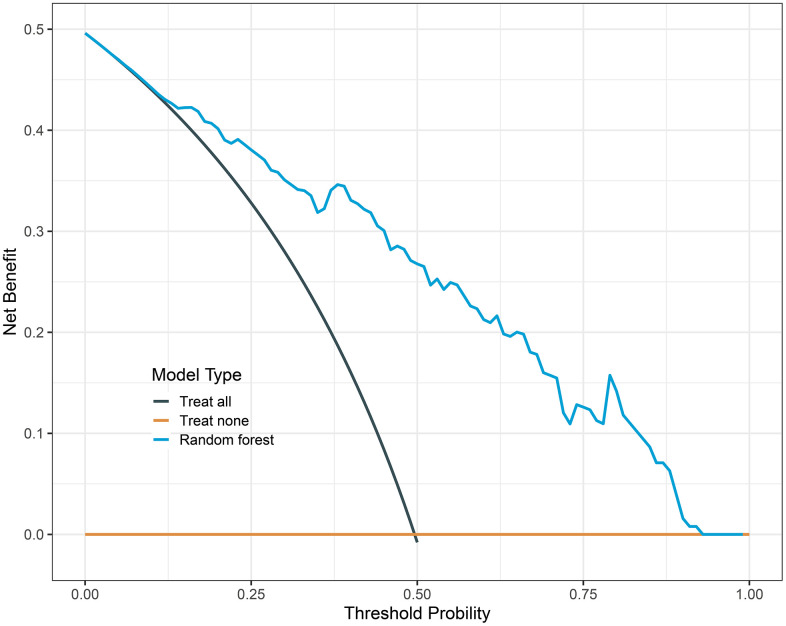
**Decision curve analysis of random forest model.** The black line is the net benefit for a strategy of treating all men; the yellow line is the net benefit of treating none. The y-axis indicates the overall net benefit, which is calculated by summing the benefits (true positive results and subtracting the harms (false positive results).

We constructed LR, XGBoost, RF, and SVM models using the training dataset. The prediction performances of these four models are presented in Table 3. When evaluated on the validation dataset, these models achieved AUCs of 0.850 (95% CI: 0.783-0.918), 0.850 (95% CI: 0.783-0.917), 0.867 (95% CI: 0.806-0.929), 0.862 (95% CI: 0.799-0.926), respectively (Figure 2). The confusion matrix and balanced accuracy of four models can be found in Supplementary Table 1. Among them, RF exhibited superior predictive performance with an AUC of 0.867 (95% CI: 0.806-0.929). Conversely, LR and XGBoost demonstrated relatively poorer generalization abilities with AUCs of only AUC of 0.850 (95% CI: 0.783-0.918) and AUC value of 0.850 (95% CI: 0.783-0.917), respectively. Therefore, we selected the RF model for subsequent analysis. Table 4 provides the detailed information regarding multivariable LR analysis. Moreover, we applied decision curve analysis to the RF model. As shown in Figure 3, the decision curve analysis demonstrated that when the threshold probability ranges from 4% to 93%, the net benefit level of applying the random forest model is significantly higher than the “Treat all” and “Treat none” strategies. This suggests that our model exhibits favorable clinical applicability.

**Table 3:** Model performance using training and validation cohorts.

**Cohort**	**Model**	**AUC (95%CI)**	**Accuracy**	**Sensitivity**	**Specificity**
Training	LR XGB	0.840 (0.795-0.884) 0.971 (0.954-0.987)	0.779 0.925	0.695 0.922	0.856 0.928
	RF	0.984 (0.974-0.995)	0.946	0.936	0.954
	SVM	0.940 (0.914-0.966)	0.881	0.936	0.830
Validation	LR XGB	0.850 (0.783-0.918) 0.850 (0.783-0.917)	0.795 0.780	0.667 0.762	0.922 0.797
	RF	0.867 (0.806-0.929)	0.780	0.714	0.844
	SVM	0.862 (0.799-0.926)	0.764	0.857	0.672

LR indicates logistic regression; SVM, support vector machine; RF, random forest; XGBoost, extreme gradient boosting.

**Table 4:** Multivariable logistic analysis for variables selected by LASSO analysis.

**Variables**	**OR (95 CI%)**	**P**
Age	1.07 (1.04-1.10)	<0.001
Treatment coiling	0.38 (0.23-0.63)	<0.001
WFNS	2.40 (1.85-3.13)	<0.001
mFS	1.55 (1.10-2.18)	0.012
Aneurysm multiple	2.56 (1.35-4.87)	0.004

WFNS, World Federation of Neurosurgical Societies; mFS, modified Fisher scale.

### SHAP model explanation

**Figure 4:**
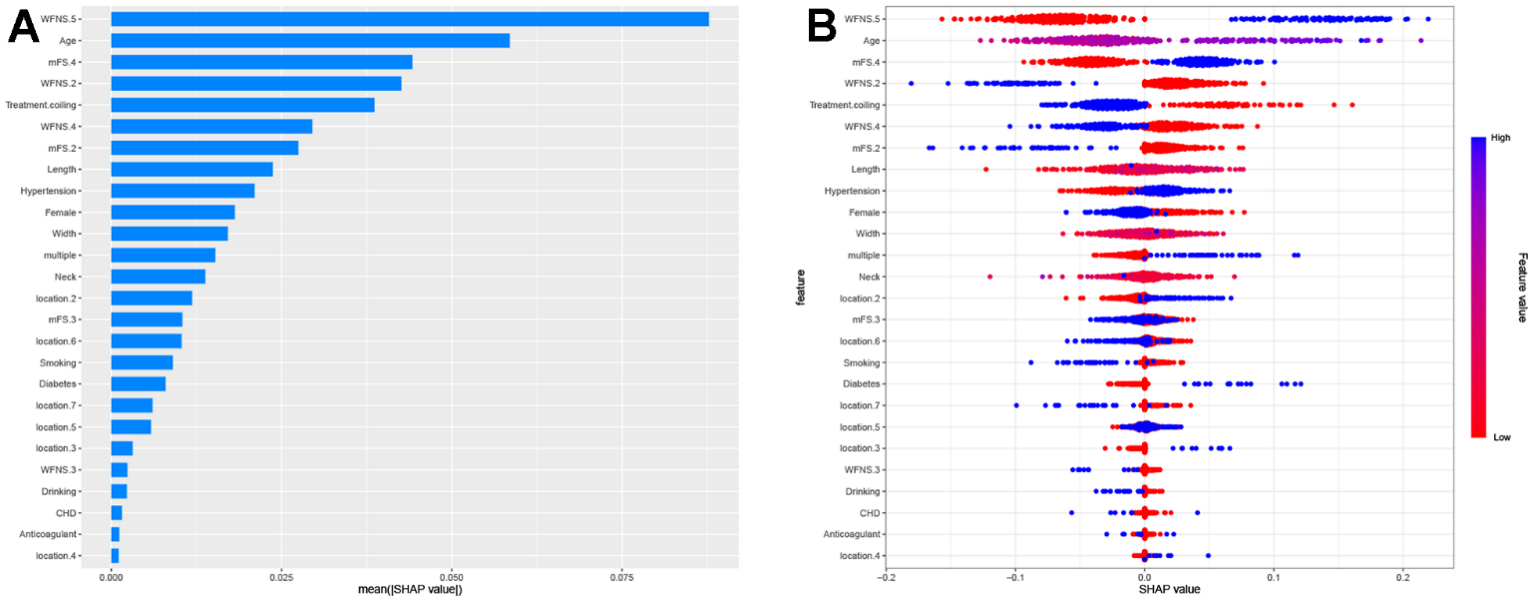
**Summary plots of SHapley Additive exPlanations (SHAP) values.** (**A**) SHAP feature importance quantified through the average absolute Shapley values. This plot illustrates the significance of each feature in development of the predictive model. (**B**) Representation of the influence exerted by each feature on the final model output, assessed via SHAP values distribution. Every individual patient is denoted by a data point within each row. The color indicates whether the continuous feature is at a high level (displayed in blue) or a low level (displayed in red) for that specific observation. When it comes to categorical features, the color blue signifies “yes”, while the color red corresponds to “no”. Location 1, 2, 3, 4, 5, 6, 7 denotes anterior cerebral artery, middle cerebral artery, internal cerebral artery, posterior cerebral artery, anterior communicating artery, posterior communicating artery and others, respectively.

The SHAP values were calculated to represent the feature importance for the RF model, which exhibited superior discriminatory capability in the validation cohort. In Figure 4A, the clinical features are ranked based on their average absolute SHAP values to showcase their relative significance. Figure 4B provides a comprehensive visualization of how factors influence the RF model, with blue indicating high level and red representing low levels for continues features in each specific observation. For categorical features, blue denotes “yes” while red corresponds to “no”.

**Figure 5:**
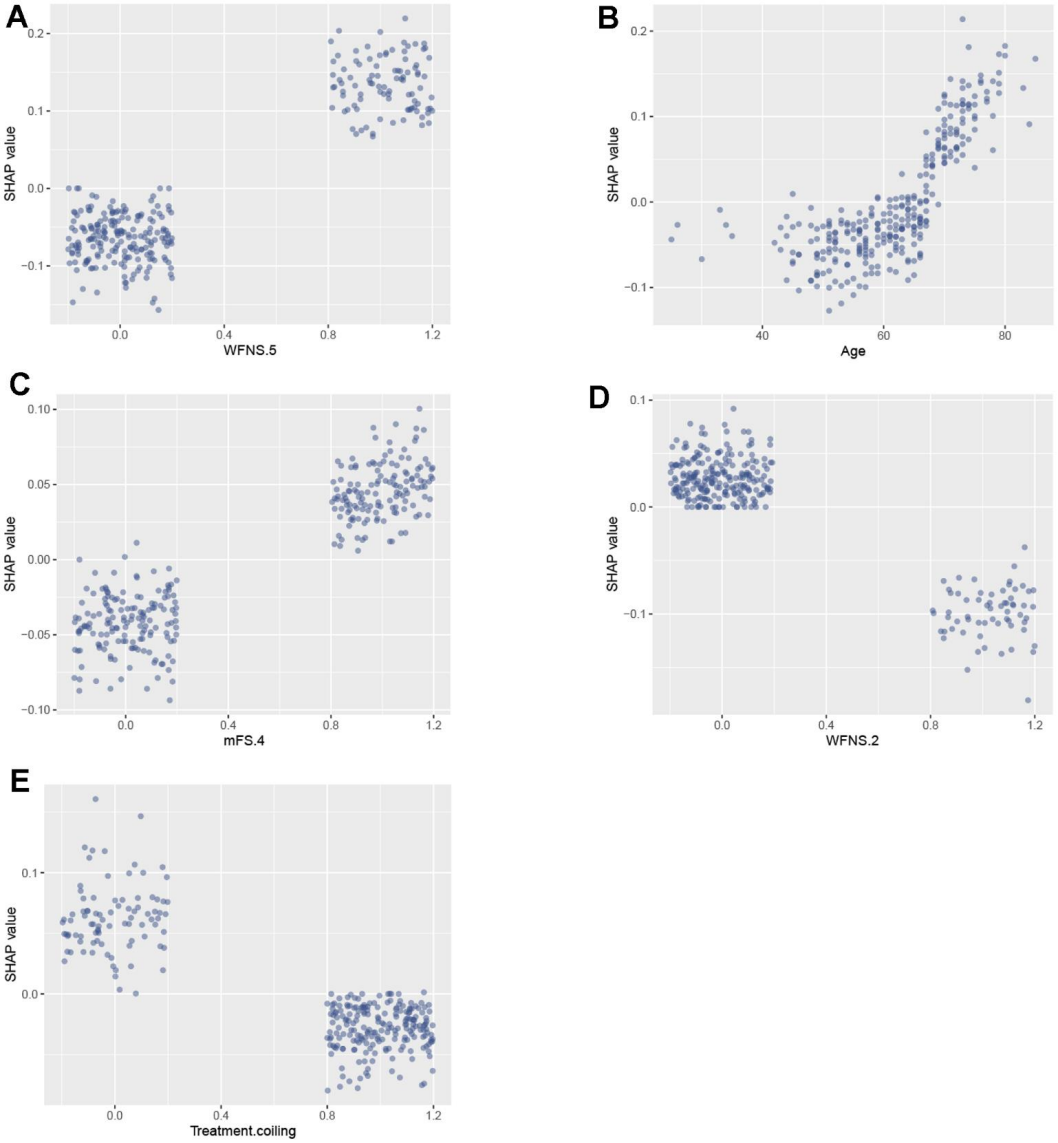
**SAHP dependency plot illustrating the top 5 clinical features in the random forest model.** (**A**) WFNS 5; (**B**) Age; (**C**) mFS 4; (**D**) WFNS 2; (**E**) Treatment coiling. WFNS, World Federation of Neurosurgical Societies; mFS, modified Fisher scale.

The features specifically associated with poor prognosis included WFNS grade 5, age, mFS grade 4, hypertension, and aneurysm multiplicity. Each of these features exhibited a positive impact and drive the prediction towards poor prognosis. Additionally, treatment of coiling embolization, WFNS grade 2, female gender exhibited a negative impact and drove the prediction towards favorable prognosis. Furthermore, we employed SHAP dependency plots to further investigate the influence of the top five factors on the RF model’s predictions of mortality risk. Figure 5 demonstrates a significant association between WFNS grade 5, mFS grade 4 and advanced age with an increased risk of poor prognosis, while treatment of coiling embolization and WFNS grade 2 are associated with a decreased risk of poor prognosis.

### Individual SHAP force plot

**Figure 6:**
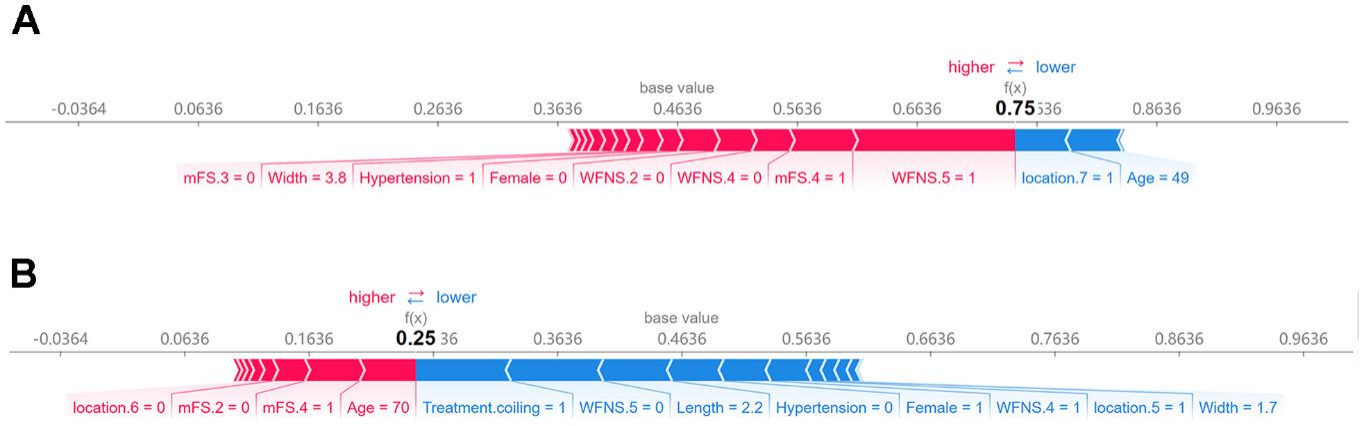
**SHAP force plot for interpreting individual’s prediction outcomes.** This plot offers a visual illustration of the RF model’s predictions, wherein the red and blue bars signify risk factors and protective factors, respectively. The length of the bars corresponds to the extent of feature importance. (**A**) Poor outcome; (**B**) favorable outcome.

The SHAP force plot analysis was employed to explain the individualized prediction outcomes of two specific samples. Figure 6 illustrates a visual representation of the predictions made by the RF model, with red and blue bars denoting risk factors and protective factors, respectively. The length of each bar corresponds to its feature importance. According to our constructed model (Figure 6A), this patient was predicted to have a 75% probability of poor prognosis. Notably, WFNS grade 5, mFS grade 4, aneurysm width of 3.8 mm, and hypertension were identified as the primary factors influencing this prediction outcome. In contrast, another patient was projected by our model to have a 25% likelihood of experiencing a poor prognosis (Figure 6B).

## DISCUSSION

In this study, we developed and validated four distinct machine learning models (LR, RF, SVM, and XGBoost). We observed that the RF model outperformed LR, SVM, and XGBoost in terms of performance (AUC=0.867, 95% CI: 0.806-0.929). To ensure both model performance and clinical interpretability, we employed the SHAP method to elucidate the decision-making process of the RF model. This effort will greatly aid physicians in gaining a comprehensive understanding of the underlying model’s decision-making process and facilitate the utilization of prediction results. The feature importance analysis revealed WFNS grade, age, mFS, and treatment of coiling embolization as predominant predictors for poor prognosis in the RF model. Our findings indicate that higher WFNS grade, higher mFS grade, and advanced age are distinct predictors for poor prognosis in high-grade SAH patients; while the treatment modality of coiling embolization serves as a protective factor.

High-grade aSAH is associated with elevated mortality and unfavorable neurologic outcomes [13]. A recent investigation revealed significant proportion of survivors of high-grade aSAH showed a good quality of life after appropriate clinical decision making [14]. Therefore, there is a critical need for early prediction of long-term functional outcomes and the identification of risk factors. Shen et al. introduced a novel scoring model for accurate prognosticate the outcomes of high-grade aSAH patients, and the model demonstrated a noteworthy AUC of 0.831 in the validation cohort [15]. Hou et al. revealed that the utilization of the neutrophil percentage to albumin ratio in constructing a predictive model for high-grade aSAH patient prognosis yielded an area under the ROC curve of 0.78 [16]. In a recent investigation on ML modeling for high-grade aSAH patient prognosis, Liu et al. reported an AUC of 0.88 achieved by their decision tree model [17]. As we can see, the predictive capability of machine learning models surpasses that of conventional predictive models. However, their model was constructed using limited algorithmic tools and lacked interpretability, functioning as a “black box” [18]. Meanwhile, explainable ML has been demonstrated successful in various medical domains such as early prognosis prediction in sepsis [19] and enabling precision medicine in acute myeloid leukemia [20]. The SHAP method introduced by Lundberg and Lee offers a game- theoretic approach that effectively addresses the black box nature of ML models [10]. Nevertheless, its application in predicting long-term prognosis for high-grade aSAH remains unexplored to the best of our knowledge. Therefore, this study represents the first attempt to employ the SHAP method within RF models for long-term prognostic prediction in patients with high-grade aSAH.

In this study, our research indicated that RF model outperforms other models in predicting the long-term prognosis of high-grade aSAH patients. Random Forest, an ensemble algorithm based on decision trees derived from random feature subsets, is widely recognized for its robust utility in feature classification and prediction tasks [21]. Moreover, RF exhibits significant advantages over other models in addressing highly non-linearly correlated data, demonstrating robustness to noise, simplicity in tuning, and facilitating efficient parallel processing [22]. Another notable strength of our study lies in the application of SHAP values, allowing us to uncover the black box of machine learning models. And the interpretable machine learning model have revealed that significant clinical variables contribute to predict the long-term prognosis of high-grade aSAH.

WFNS grade, a widely recognized classification schema for assessing the severity of aSAH, categorizes patients into five grades based on clinical neurological manifestations [23, 24]. Higher WFNS grades often associate with more profound neurological deficits and poorer clinical outcomes [25]. Bogossian et al. found that patients with high-grade aSAH contribute to have significant rates of poor prognosis, particularly those classified as WFNS grade 5 upon admission [26]. Another study revealed that even with prompt intervention, patients with WFNS grade 5 exhibited a prevalence of severe disability at discharge reaching 27% [27]. Our finding in the context of high-grade aSAH consistent with the well-established understanding that higher WFNS grade correlate with worse prognosis, which can be attributed to aggravated neurological impairment and increased risk of subsequent complications [28, 29].

Advanced age was positively correlated with higher WFNS grade, and the older the patient, the higher the probability of presenting in a deteriorated condition after aSAH [30]. Previous studies have revealed advanced age as independent predictors of poor prognosis in patients with high-grade aSAH [25, 31, 32]. Advancing age causes patients more susceptible to cerebral insults, diminishes physiological reserves, and impairs recovery mechanisms [33, 34]. Elderly patients often suffer increased burdens of comorbidities and declined physiological recovery capacity, resulting them being susceptible to unfavorable outcomes following high-grade aSAH [25, 35].

mFS system, a radiological tool assessing hemorrhage extent on computed tomography scans, plays a key role in evaluating the severity of bleeding and vasospasm risk [36]. Similar to the WFNS grade, a higher mFS score indicates more extensive hemorrhage and are linked to unfavorable outcomes [15]. The severity of bleeding and its subsequent complications such as vasospasm and delayed cerebral ischemia contribute to the observed correlation between elevated mFS score and poor prognosis in high-grade aSAH [15, 17, 37].

Endovascular coiling, a minimally invasive strategy for the treatment of intracranial aneurysm, represents a promising technique for high-grade aSAH patients [38, 39]. Recent years have witnessed a significant advancement in the prognosis of patients with high-grade aSAH, with rates of functional independence ranging from 30%-57% [40, 41]. These improved outcomes have been attributed to the early and aggressive implementation of endovascular coiling [42, 43]. However, another recent study indicated that, in comparison to surgery being a short-term morbidity risk factor, endovascular treatment is associated with higher mortality rates at 1 year [44]. The observed findings are probably a consequence of selection bias inherent in the retrospective nature of the data. In general, through the effective occlusion of aneurysms with metal coils, the risk of rebleeding and subsequent complications can be attenuated.

In summary, our study established four ML models (LR, SVM, RF, XGBoost) and selected the RF model to conduct a comprehensive SHAP analysis based on its superior predictive performance. The SHAP analysis revealed the significant contributions of clinical features in predicting long-term prognosis in high-grade aSAH. Elevated WFNS grades and mFS, along with advanced age, were associated with unfavorable outcomes, indicating aggravated neurological impairment and bleeding severity. Conversely, the strategic implementation of endovascular coiling emerges as a promising method to improve patient prognosis by preventing rebleeding and mitigating associated complication. Incorporating these insights into clinical decision-making holds great potential to guide therapeutic strategies and optimize patient neurocritical care. Moreover, the predictive model we developed paves the way for personalized treatment strategies. Patients identified as having an elevated risk of unfavorable outcomes according to our model could gain benefits from intensive monitoring, early intervention, and personalized rehabilitation approaches. In summary, the explainable ML models serve as a valuable tool to improve clinical decision-making regarding the prognosis of high-grade aSAH.

However, this study also had several limitations. Firstly, the single-center design may limit the generalizability of findings to broader patient populations, and an independent validation cohort from other centers for model evaluation is necessary. Secondly, there might be several unobserved confounders that could potentially influence the prognosis outcomes of high-grade aSAH patients. Thirdly, the lack of external validation data from other medical center will further restrict the model’s generalizability, thus additional prospective randomized clinical trials are essential to validate our model. It should be noted that our modeling study exclusively enrolled adult patients, leaving the predictive validity of the RF model for pediatric high-grad aSAH remains unclear.

## CONCLUSIONS

In this study, we employed four machine learning algorithms and identified the Random Forest (RF) model as the most effective predictor of long-term prognosis for high-grade aSAH patients. By utilizing SHAP analysis, we highlighted the crucial role of key variables such as WFNS grade, modified Fisher score, age, and endovascular coiling, in prognosis determination. Our approach not only ensures precise prognostic predictions, but also enhances transparency and interpretability of clinical decisions, thereby leading to improved patient outcomes. Ultimately, our study highlights the significance of RF and SHAP in enhancing prognostic accuracy and guiding personalized care for high-grade aSAH patients.

## MATERIALS AND METHODS

### Study design and participant enrollment

This single-center, prospective registered (NCRIA-1: NCT05738083), observational cohort study was conducted at Department of Neurosurgery, The Second Affiliated Hospital of Nanchang University between October 2018 to December 2021. In accordance with the guidelines, aSAH diagnosis was established through computed tomography (CT), CT angiography, or digital subtraction angiography. The inclusion criteria were defined as follows: (1) spontaneous aneurysmal subarachnoid hemorrhage; (2) hospital admission within 72 hours of symptom onset; (3) Hunt and Hess grade of III and V; (4) non-contrast CT scan performed upon admission; (5) receipt of aneurysm treatment within 72 hours after onset; (6) documentation of postoperative complications and mortality; and (7) corresponding follow-up records.

Exclusion criteria included the presence of vascular malformation or other cerebrovascular disease, postoperative status at admission, permanent brain injury at presentation, death within 3 days after operation, and missing data.

### Prediction variables collection

The following variables were collected from the hospital electronic health record system: (1) patient demographic information, including sex, age, and past medical history such as hypertension, diabetes mellitus, coronary heart disease, smoking habits, alcohol consumption patterns, and anticoagulant usage; (2) admission clinical status indicators encompassing World Federation of Neurosurgical Societies (WFNS), Hunt and Hess grade (HH), and modified Fisher scale (mFS); and (3) aneurysm details comprising location, number, length, width, neck size as well as treatment modality.

### Definition of outcome

The neurological outcome of these patients was evaluated at 12 months after initial aSAH using modified Rankin scale (mRS) system [45, 46]. A favorable neurological outcome was defined as mRs 0 to 2, while a poor outcome was considered when mRs ranged from 3 to 6. The patient follow-ups were conducted by a neurosurgeon through telephone consultations. And the neurosurgeon was blinded to the patients’ clinical information.

### Machine learning model development

Each patient with high-grade aSAH was considered as an individual data point in our dataset. The clinical information assessed at admission, including demographic data, past medical history, WFNS grade, mFS, aneurysm details and treatment modality, was utilized as features to predict the 12-month prognosis. Prior to model training, categorical variables were subjected to one-hot encoding to guarantee consistent and effective utilization of dataset. Through this method, categorical variables were converted into binary matrix, effectively eliminating ordinality and preventing inadvertent hierarchical structures.

The dataset was divided into training and validation sets in a 7:3 ratio, and the training set was used to develop four ML models, including the logistic regression model (LR), support vector machine (SVM), random forest (RF), and extreme gradient boosting (XGBoost). Moreover, to avoid overfitting in LR, the features were selected and filtered by the Least Absolute Shrinkage and Selection Operator (LASSO) model with the “λ-1se” criterion. Each algorithm was fine-tuned through hyperparameter optimization to optimize model performance. The ten-fold cross-validation was employed to mitigate any potential bias in the data and ensure the generalization of model performance.

### Feature importance with Shapley Additive Explanation values

The Shapley Additive Explanation was employed to enhance the interpretability of the final model. In cases where the SHAP value is positive, it suggests that the associated feature contributes to an increased risk of the complications. Meanwhile, a negative SHAP value indicates that the corresponding feature is linked to a decreased risk of the complications. The magnitude of SHAP values signifies the extent of a feature’s contribution to the prediction performance.

The SHAP summary plot was utilized to demonstrate the contributions of each feature attributed to the model. Besides, the SHAP force plot was used to visualize the effects of pivotal features on the final model for individual patients. The “fastshap” package in R software was used to analyze the SHAP values, the “ggbeeswarm” and “shapviz” packages were used to visualize the SHAP values for each feature.

### Statistical analysis

Before conducting the formal analysis on the dataset, the Kolmogorov-Smirnov test was employed to ascertain the distribution type of the data. Continuous variables were analyzed using the independent t-test or Mann-Whitney U-test and were reported as a median with interquartile ranges or mean ± SD. For categorical variables, the Chi-square test or Fisher’s exact test was used for analysis, and were represented as frequencies. The performance of the models was evaluated using the following statistical parameters: true positives (TP), false positives (FP), false negatives (FN), true negatives (TN), sensitivity, specificity and the accuracy of the models. Besides, the area under the receiver operating characteristic curve (AUC-ROC) with 95% CI and the balanced accuracy were also used to evaluate model performance. We calculated these statistical parameters on both training and validation set to show the generalization capability of these models. A two-tailed P <0.05 was considered to have a statistical significance. All statistical analyses were conducted using SPSS (Version 26.0, IBM Corp., Armonk, NY, USA) and R software (Version 4.3.0).

### Data availability statement

Anonymized data are available upon reasonable request.

## Supplementary Material

Supplementary Table 1
