# Supplementary material for: Explainable machine learning in outcome prediction of high-grade aneurysmal subarachnoid hemorrhage
Source: Aging (Albany NY). 2024 Mar 1;16(5):4654–69. doi: 10.18632/aging.205621 (PMC10968679; doi:10.18632/aging.205621)
Supplement: Supplementary Table 1 [file aging-16-205621-s001.pdf]

SUPPLEMENTARY TABLE

Supplementary Table 1. Confusion matrix of ML and LR models using training and validation cohorts.

Cohort	Model	TP	FN	TN	FP	Ba-Acc
Training	LR	98	43	131	22	0.776
	XGB	130	11	142	11	0.925
	RF	132	9	146	7	0.945
	SVM	132	9	127	26	0.883
Validation	LR	42	21	59	5	0.794
	XGB	48	15	51	13	0.779
	RF	45	18	54	10	0.779
	SVM	54	9	43	21	0.765

LR indicates logistic regression; SVM, support vector machine; RF, random forest; XGBoost, extreme gradient boosting; TP, true positive; FN, false negative; TN, true negative; FP, false positive; Ba-Acc, balance accuracy.
